# Real-Time Detection of Concealed Threats with Passive Millimeter Wave and Visible Images via Deep Neural Networks

**DOI:** 10.3390/s21248456

**Published:** 2021-12-18

**Authors:** Hao Yang, Dinghao Zhang, Shiyin Qin, Tie Jun Cui, Jungang Miao

**Affiliations:** 1 School of Electronics and Information Engineering, Beihang University, Beijing 100191, China; hbhszdh@buaa.edu.cn (D.Z.); jmiaobremen@buaa.edu.cn (J.M.); 2 School of Automation Science and Electrical Engineering, Beihang University, Beijing 100191, China; qsy@buaa.edu.cn; 3 State Key Laboratory of Millimeter Waves, School of Information Science and Engineering, Southeast University, Nanjing 210096, China; tjcui@seu.edu.cn

**Keywords:** deep learning, millimeter wave imaging, image fusion, object detection

## Abstract

Passive millimeter wave has been employed in security inspection owing to a good penetrability to clothing and harmlessness. However, the passive millimeter wave images (PMMWIs) suffer from low resolution and inherent noise. The published methods have rarely improved the quality of images for PMMWI and performed the detection only based on PMMWI with bounding box, which cause a high rate of false alarm. Moreover, it is difficult to identify the low-reflective non-metallic threats by the differences in grayscale. In this paper, a method of detecting concealed threats in human body is proposed. We introduce the GAN architecture to reconstruct high-quality images from multi-source PMMWIs. Meanwhile, we develop a novel detection pipeline involving semantic segmentation, image registration, and comprehensive analyzer. The segmentation network exploits multi-scale features to merge local and global information together in both PMMWIs and visible images to obtain precise shape and location information in the images, and the registration network is proposed for privacy concerns and the elimination of false alarms. With the grayscale and contour features, the detection for metallic and non-metallic threats can be conducted, respectively. After that, a synthetic strategy is applied to integrate the detection results of each single frame. In the numerical experiments, we evaluate the effectiveness of each module and the performance of the proposed method. Experimental results demonstrate that the proposed method outperforms the existing methods with 92.35% precision and 90.3% recall in our dataset, and also has a fast detection rate.

## 1. Introduction 

**Figure 1:**
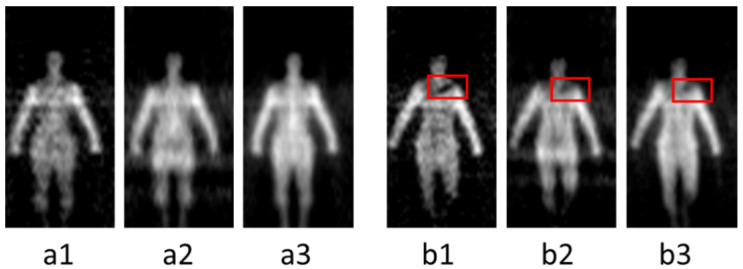
PMMWIs acquired from our PMMWI imager. (**a1**–**a3**) shows human bodies without threats; (**b1**–**b3**) are human bodies with threats (a knife), marked within red boxes. In each scene, the images are obtained by A, B2 and B3 from left to right.

The millimeter wave band is a part of the electromagnetic spectrum ranging from 30 GHz to 300 GHz. It has obtained widespread attention to the security screening due to a good penetrability to clothing and all-weather imaging features [1,2,3]. Millimeter wave can find concealed threats behind the clothing through temperature distribution which is generated by electromagnetic radiation from the target. According to different imaging mechanisms, there are two kinds of techniques: active millimeter wave imaging (AMMWI) and passive millimeter wave imaging (PMMWI) [4,5]. The AMMWI emits specific millimeter waves to the target, and the image is carried out by the synthesis of the reflected waves. The AMMWI can provide more detailed information and better image quality; however, active millimeter waves have radiation on human and thus, AMMWI is less used in practical application. The PMMWI does not produce radiation to the human as it collects radiation from human to process. Meanwhile, it has higher imaging rate than the AMMWI [6]. Many PMMWI systems have been developed [7,8,9] recently. Beihang University has been engaged into the research of PMMWI during the last few decades, and several generations of imagers of PMMWI have been developed: BHU-2D [10], BHU-2D-U [11], and BHU-256 [12]. These prototypes have demonstrated the capability of detecting threats with the advantages of high imaging rate and large field of view (FOV). To further enhance the radiometric sensitivity and spatial resolution in real-time application scenarios, the latest generation BHU-1024 with 1024 antenna-receiver channels working in Ka band (32 GHz~36 GHz) has been proposed. This system is capable of fast imaging (25 frames per second), and the correlation system, which measures the visibility function through performing correlation between two signals, is achieved by digital circuits and analog circuits, respectively [13,14,15,16]. The analog correlation system (termed as A) utilizes the entire 4 GHz bandwidth to achieve high radiometric sensitivity. The digital correlation system has high integration. In our imager, two digital correlation systems are employed, which exploited 33–34 GHz (denote as B2) and 34–35 GHz (denote as B3) bandwidths. The PMMWIs obtained from this system are shown in Figure 1, and the concealed threats were marked within red boxes in each image. It could be concluded that PMMWI has the following characteristics: noise interference and incomplete shape of target. Since not all low-gray regions are related to threats, the thresholding method might be infeasible.

In this study, our motivation was to leverage the merits of our PMMW imagery which provides multi-type images in real-time. We employed image fusion for multi-source PWWMIs to obtain a better image quality compared with existing PWWMI datasets. Aiming to overcome the high false alarms caused by detection only by PMWWIs, we developed strategy to explore the advantages of visible imagery (VI) collected by Kinect; the Kinect is equipped in our imagery and has the same imaging rate as PMMWIs. With the incorporation of multi-source PMWWIs and VI, we designed a multi-stage detection method to achieve improved detection accuracy. First, a GAN-based [17] network was designed in this study for its superiority in extracting the most discriminate feature in source images and merge it into a single fused image in an unsupervised manner. We then exploited it to fuse the PMMWI obtained from A (analog PMMWI) and B3 (digital PMMWI) for the purpose of image enhancement. Second, due to the outstanding performance of UNet [18] in medical image segmentation, we employed a TernausNet [19] that is a version of UNet to adapt different domains (PMMWI and VI) by utilizing pre-training weights. Combining with the transfer learning [20], we developed an improved TernausNet to fuse the shallow semantic information and deep details in the fused PMMWI and VI via a top-down manner to extract the region of threats. Third, an end-to-end network is proposed for image registration which can calibrate the disparities of scales and FOV between PMMWI and VI to further locate the threats and preclude the false alarms. The subsequent inspections employing the registered PMMWI/VI determine the region of threats and eliminate false alarms. In the last stage, two separate networks were used for metallic and non-metallic detection and a multi-frame analysis was conducted based on the results of each single frame. 

The main contributions of the proposed method are outlined as follows:

- The combination of VI and PMMWI. The combination of VI and PMMWI lies in two aspects. First, the weights of our segmentation network are shared between PMMWI and VI except for the batch normalization layer [21]. The combination of PMMWI and VI accelerates the training process of network and improve the segmentation effects. Second, the false alarm can be excluded by a detection method that combined the high penetrability of PMMWI and the high resolution of VI. The experimental results show that our method improves the accuracy and robustness of the detection.
- The fusion of multi-source PMMWIs. A GAN-based network is developed to achieve the fusion of digital PMMWI and analog PMMWI, which can generate the images with higher contrast and SNR. Our method introduces multiple image information to overcome the defects of denoising techniques [22]. Meanwhile, the proposed network is lightweight and easy for training.
- A multi-stage detection pipeline is proposed. Through the fusion and segmentation stages, imaging quality and the accuracy of detection are improved. Moreover, the non-metallic threats can be also identified from human body by contour information, to which few researches have referred. Additionally, the efficiency of the detection is enhanced, for the method facilitated the detection of a non-stationary manner through inspection channels.

## 2. Related Works

Many techniques have been proposed and applied in the PMMWI system. Traditional methods are mainly based on classic machine learning theory or statistic, where hand-designed features are indispensable for recognition of threats. In [23], a multi-level thresholding method based on Otsu’s algorithm is adopted to achieve auto-classification and segmentation for detection of metallic threats in PMMWI. K-means clustering and expectation maximization (EM) algorithm [24] are employed to segment the body area and concealed threats successively. The wavelet fusion and the sum of squared difference (SSD) [25] are utilized to extract the most relevant frames from the sequences of PMMWIs, improving the robustness of the detection. However, hand-designed features are based on the low-level features of threats, and have limited representative capabilities. The hand-designed features are suitable for few scenarios, and lack robustness. To achieve a better detection, traditional methods also attempt to introduce image enhancement technique into the PMMWI detection system. An adaptive manifolds filtering algorithm [26] is proposed for improving the quality of PMMWI, and then the kernel support vector machine is adopted to classify threats. Deconvolution [27], as another image enhancement technique, approximates the posterior distribution by a Dirichlet distribution and this approach achieves the effect of noise removal. These methods show an acceptable denoising effect in a specific environment. However, the characteristics of PMMWI change with different time, temperature, and environment, etc., and it is difficult to define a denoising method in varying environment in advance. 

Since the recent decades, the deep learning-based method has been superior to aforementioned traditional machine learning methods based on low-level vision features in many fields, such as object detection, human face recognition, and semantic segmentation. Deep learning is able to extract high-level discriminative semantic features, which are conducive to identify the threats in complex scenes. Some work has been done to introduce deep learning into threat detection in the PMMWI. Deep CNNs architecture [22,28] is proposed to detect multiple types of concealed threats in PMMWI. In [29], YOLOv3 is adopted for localization and the recognition of the concealed threats with a high speed. Cheng et al. [30] employs an improved maximum entropy algorithm to increase the accuracy of threat segmentation, and in the classification stage, a CNNs with Inception Module is designed to achieve good performance on the dataset. The results show that the method can cope with the non-stationary noise existing in the PMMWI, and the detection rate is greatly improved compared with the traditional methods. Despite some progress in recent study, the methods above only focus on PMMWI for detection, but due to a lower resolution and SNR, the information contained in PMMWI can be corrupted by noise. Unfortunately, these methods are not robust enough to reveal the high-level semantic features with the interference of noise. Other work attempts to tackle this issue by combining the advantages of VI. Li et al. [31] extracts the contour feature of threats with the employment of pulse-coupled neural network for the fusion of VI and PMMWI in Tetrolet domain. In [32], two networks are developed for human body profile segmentation on PMMWI and VI separately, and the suspected targets are localized by registrating the segmented PMMWI and VI. Although these methods adopt VI to assist the detection, and have obtained certain progress, the methods above concern with each single frame of images, and it is difficult to obtain reliable results using the only single frame. In addition, the existing methods fail to distinguish non-metallic threats based on grayscale features. Different from the reviewed methods, this paper makes a comprehensive utilization of multi-source PMMWIs and VIs for simultaneous detection of metallic and non-metallic threats in real time when a person moves through the inspection channel in front of the imager. In practical application, the influence of clothing texture, gender, and posture are also included. 

The rest of this paper is organized as follows. The illustration of the proposed method is presented in Section 3. Section 4 illustrates the experimental setup and experiment results. The conclusion is presented in Section 5. 

## 3. The Proposed Method

**Figure 2:**
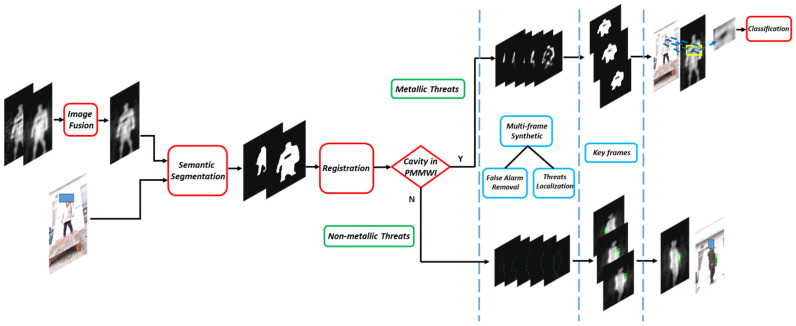
The flow diagram of the proposed solution. The detection process consists of 4 stages: First, analog and digital PMMWIs are fused to obtain high-quality PMMWI. Then the fused PMMWI and VI are segmented to extract human body and threats. The size of human body in the segmented images is adjusted to similar scale through registration network. In the comprehensive analysis stage, the detection is divided into two branches: metallic threats and non-metallic threats. Each branch performs detection on single frame, and then the proposed multi-frame synthesis strategy is applied on every 5 consecutive frames to produce a key frame. Finally, the IOU between two areas is calculated in key frames for each person and select the largest one as the finally result. The metallic threats will further to be identified by the classification network.

In this paper, we propose a novel real-time solution for detecting the concealed threats in human body. The schematic of this solution is shown in Figure 2. The whole solution can be divided into four sub-modules: 

Image fusion: As illustrated in Section 1, we can observe that the analog PMMWI has higher sensitivity, and the digital PMMWI remains smooth and can reveal complete information of the contour of body and threats. It is intuitive to synthesize both types of PMMWI for better imaging quality. We propose a GAN based architecture [17] to extract features from each type of PMMWI in an unsupervised manner, which can be used to reconstruct PMMWI. 

Semantic Segmentation: We deploy this module to identify the regions of human body and threats in the PMMWI. We introduce a separate batch normalization layer into the TernausNet. This layer allows different batch normalization parameters for different types of input images, i.e., PMMWI/VI, while the other parts of the network share the same parameters.

Image Registration: The reason we apply image registration lies in two aspects: locating the threats and precluding false alarms. Both VI and PMMWI can provide information of human body; however, due to the different scales and FOV, the human body region in the two images cannot be directly compared. Fortunately, we can find the correspondence between locations in the VI and PMMWI through the registration, and further, we can locate the threats and remove false alarms by comparing the registered images. Thus, we propose a network to extract similar sub-regions existing in PMMWI and VI, and the loss function is also designed to achieve efficient unsupervised learning. Moreover, the visualization of detection results in the VI is indispensable in the real application; this issue will be solved by the correspondence between VI and PMMWI.

Threats Detection: In this module, the detection is divided into two pipelines: (1) Localization and classification for metallic threats. (2) Localization for non-metallic threats by proposing an anomaly area detection network deriving from PointNet [33] to explore the contour feature of human body and determine the abnormal contour area caused by threats. We integrate the detection results of metallic and non-metallic threats in each frame and identify metallic threats through a classification network.

### 3.1. Image Fusion Network

The key to image fusion is the process of feature extraction from source images and the mergence into a single fused image. The fused image preserves salient features, and the redundant information is removed. 

**Figure 3:**
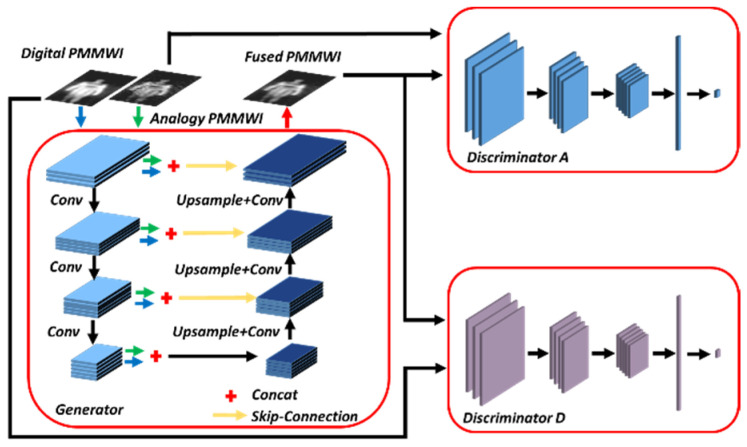
Architecture of the fusion network contains one Generator and two Discriminators.

In fusion methods based on traditional GAN, there is no control on modes of the data being generated, the fused image is trained to be similar to only one of the source images, leading to the loss of some information contained in the other source image [34]. However, by conditioning the model on additional information it is possible to direct the data generation process. The GAN can be extended to a conditional model if both the generator and discriminator are conditioned on some auxiliary information, such as class labels or data from other modalities [35]. Consequently, we propose a conditional GAN-based [36] architecture, and according to [34], two discriminators and a generator are employed. Given one analog PMMWI $I_{a}\in {\mathbb{R}}^{160\times 80}$ and one digital PMMWI $I_{d}\in {\mathbb{R}}^{160\times 80}$, we aim to learn a conditional generator $G\left(I_{a},\;I_{d}\right)$ (conditioned on $I_{a}\;\mathrm{and}\;I_{d}$) which generates fused image to fool the discriminator. Two discriminators $D_{a}$ and $D_{d}$ are applied to distinguish the fused images from source images $I_{a}$ and $I_{d}$ to produce a scalar output indicating whether the fused image belongs to the real one or G. Figure 3 presents the image fusion network.

Generator Architecture: Our generator is similar to UNet [18]. It contains encoder, feature fusion layer and decoder. The networks for $I_{a}$ and $I_{d}$ share the same weights. The encoder consists of 4 convolutional layers with 3 × 3 filters and the number of output channels gradually increases when high-level features are extracted. In this paper, they are 16, 28, 40, and 52 respectively. Then, we concatenate the corresponding outputs for each convolution layer of inputs $I_{a}$ and $I_{d}$. The decoder also consists of 4 convolutional layers. With the help of skip-connections [37], the shallow features of the encoder are added in the decoder for the reconstruction of fused image $I_{f}\in {\mathbb{R}}^{160\times 80}$. This structure also has the advantage of reducing computational complexity.

Discriminator Architecture: In the view of the similarity between $I_{a}$ and $I_{d}$, the stronger one in $D_{a}$ and $D_{d}$ will affect the performance of the other. Thus, we design $D_{a}$ and $D_{d}$ with the same structure. Due to different imaging mechanisms of $I_{a}$ and $I_{d}$, the discriminator will retain different weights after training process. The discriminator includes 3 convolutional layers with 3 × 3 filters, and can obtain 16, 32, and 64 feature maps respectively. Followed by convolutional layers, a fully connected layer is employed to produce the probability that indicates the source of the input images.

Loss Function: For the generator, we hope that the generated image retains the characteristics of $I_{a}$ and $I_{d}$ as much as possible; therefore, we introduce structure similarity loss $L_{ssim}$ and total variation loss $L_{TV}$ [38] into the generator loss function:

(1) $$L_{Gen}=L_{Gen}^{ori}+\lambda L_{TV}+\gamma L_{ssim}$$

where the hyperparameters λ and γ are trade-off factors, and $L_{Gen}^{ori}$ the original GAN loss function.

It can be seen from Figure 1 that the $I_{d}$ has more intact contour information of human, and it is important to subsequent detection and visualization. We employ $L_{pixel}$ to achieve similar pixel intensities between $I_{d}$ and fused image $I_{f}$, the $L_{pixel}$ is expressed by

(2) $$L_{pixel}=\|I_{d}-I_{f}{\|}_{F}^{2}$$

where $\|\cdot {\|}_{F}$ is Frobenius norm.

The $L_{TV}$ reflects the gradient difference between two images. We observe that concealed threats have higher contrast in $I_{a}$. Consequently, we add $L_{TV}$ term to retain gradient information of $I_{a}$ in the fused image $I_{f}$. The $L_{TV}$ is calculated by

(3) $$L_{TV}=\sum_{i,j}\;\left(\|R\left(i,j+1\right)-R\left(i,j\right){\|}_{F}+\|R\left(i+1,j\right)-R\left(i,j\right){\|}_{F}\right)$$

(4) $$R\left(i,j\right)=I_{f}\left(i,j\right)-I_{a}\left(i,j\right)$$

where R is difference between $I_{a}$ and $I_{f}$.

In this paper, the loss function of discriminators follows the original form in [17], and its function is to guarantee the distribution of the generated data close to that of the original data.

### 3.2. Semantic Segmentation Network

The VI and PMMWI are from different domains (distributions), while they still have common features, i.e., body shape. Therefore, we employ a network based on TernausNet. The network extracts the common features of VI and PMMWI through sharing part of the parameters, and the separate batch normalization layer learns the unique features of VI/PMMWI. The network attains a trade-off between computational efficiency and accuracy. 

Network Architecture: The TernausNet is a fully convolutional network, and its structure is also divided into two parts: the encoder and the decoder, which is similar to that of generator in the image fusion part. To improve the ability of feature extraction, its encoder adopts the same structure of VGG [39], so the pretrained VGG on large datasets (e.g., ImageNet) can be utilized. The decoder consists of convolutional layer and deconvolutional layer, and the former is used for channel fusion while the latter is intended for up-sampling. The cross-layer connection structure is used between the encoder and the decoder; hence, the features with the same resolution are concatenated in the channel dimension to achieve the fusion of high-level and low-level features.

**Figure 4:**
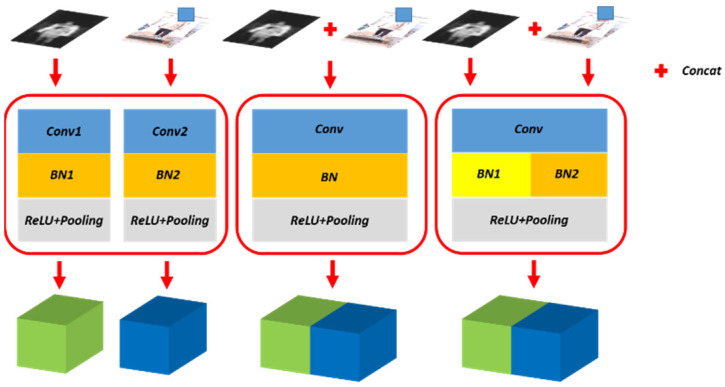
Illustration of the proposed separate batch normalization layer. The left shows two segmentation networks for PMMWI and VI respectively, and the middle employs one network for PMMWI and VI segmentation. The right is our method. We introduce two independent BN layers to learn PMMWI and VI features respectively, while the remaining of the network is shared.

Separate Batch Normalization: The batch normalization layer reflects the distribution of current data. Through the batch normalization layer, the output of different layers acquires similar distribution, and the versatility of the feature is enhanced consequently. We propose separate batch normalization layer, which uses different parameters for different data domains, i.e., the whole network except for the batch normalization layer share same parameters. In this way, although the data has different distributions, the different batch normalization layer parameters ensure that they have a similar distribution after normalization, which makes the training process of the feature extraction easier and enhances the versatility of feature extraction. The schematic diagram of separate bath norm layer is shown in Figure 4.

Loss Function: In the training phase, we apply Binary Cross Entropy as the loss function:

(5) $$L_{seg}=-\frac{1}{N}\sum_{i=1}^{N}\;\left[y_{i}\;log\left(x_{i}\right)+\left(1-y_{i}\right)log\left(1-x_{i}\right)\right]$$

where N is the number of pixels, $x_{i}$ refers to the value of ith pixel in the predicted image, and $y_{i}$ is the corresponding groundtruth.

### 3.3. Image Registration Network

The original PMMWI/VI is non-homogenous; however, after being segmented, they are homogenous, which are put into our registration network. The PMMWI/VI in our system captured simultaneously from the same imaging angle. Therefore, we employ 4 degrees of freedom to quantify the transformation parameters: horizontal scale, vertical scale, horizontal offset and vertical offset. 

**Figure 5:**
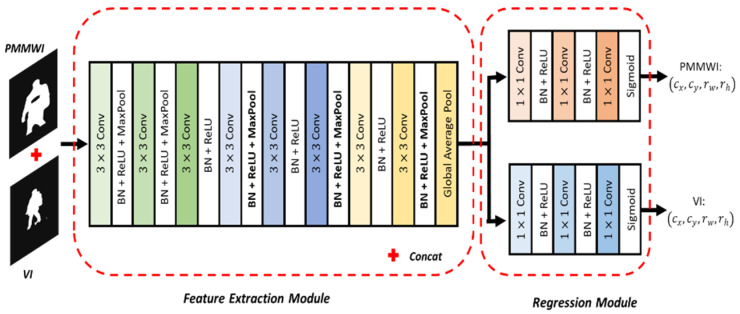
Framework of the proposed registration network.

Network Architecture: Our registration network is shown in Figure 5. The network includes feature extraction module and regression module to calculate 4-dimensional parameters of image transformation. The fully-connected layer of VGG11 [40] is removed to function as the feature extraction module. The feature extraction module is shared between the two image inputs. Then we add two separate branches for the regression of transformation parameters. The two regression branches have the same architecture with 3 fully-connected layers, where the last layer outputs a 4-dimensional vector representing the transformation parameters. The first two layers use Rectified Linear Unit (ReLU) as the activation function, and the last layer uses the Sigmoid as the activation function. We denote the outputs of the fully-connected network as $\left(c_{x},\;c_{y},\;r_{w},\;r_{h}\right)$, and they range from 0 to 1. However, this parameter may cause the sub-region to exceed the original image. Therefore, we perform the following conversion:

(6) $$\{\begin{array}{c} d_{w}=r_{w}\\ d_{h}=r_{h}\\ d_{x}=(1-r_{w})c_{x}\\ d_{y}=(1-r_{w})c_{x} \end{array}$$

where $d_{w}$ and $d_{h}$ refer to the scaling ratio in width and height, respectively; $d_{x}$ and $d_{y}$ are offsets in width and height, respectively. After conversion, the transformation parameters satisfy the following:

(7) $$\;\;\;\{\begin{array}{c} 0<d_{x}<1\\ 0<d_{y}<1\\ 0<d_{x}+d_{w}<1\\ 0<d_{y}+d_{h}<1 \end{array}$$

Hence, we guarantee that the sub-region is completely contained in the original image. Then we use these parameters to extract the sub-region from the PMMWI and VI, and Algorithm 1 illustrates this extraction process.

**Algorithm 1** Extraction of the similar sub-region**Input:** original image $I_{ori}$ with width W and height H,
transformation parameters $\left(c_{x},\;c_{y},\;r_{w},\;r_{h}\right)$
**Output:** sub-region $I_{sub}$
1. Calculate the transformation parameters $\left(d_{x},\;d_{y},\;d_{w},\;d_{h}\right)$ by (6)

**- Coordinate of sub-region in normalized Coordinate System:**
2. Resize the sub-region: top-left: (0, 0), bottom-right: $\left(d_{w},\;d_{h}\right)$
3. Add offset: top-left: $\left(d_{x},\;d_{y}\right)$, bottom-right: $\left(d_{w}+d_{x},\;d_{h}+d_{y}\right)$

**- Coordinate of sub-region in**

$I_{ori}$

**:**
4. Project to $I_{ori}$: top-left: $\left(d_{x}\times W,\;d_{y}\times H\right)$, bottom-right: $(\left(d_{w}+d_{x}\right)\times W,\left(d_{h}+d_{y}\right)\times H)$5. Extract the sub-region $I_{sub}$ from $I_{ori}$


Loss Function: Our goal is to extract similar sub-regions from PMMWI and VI according to the image similarity criterion. Therefore, we employ the mean square error (MSE) as the loss function. We denote $I_{1}$ and $I_{2}$ as the extracted regions from PMMWI and VI, and the similarity loss $L_{simi}$ is formulated as:

(8) $$L_{simi}=\frac{1}{mn}\sum_{i=0}^{m}\;\sum_{j=0}^{n}\;{\left(I_{1ij}-I_{2ij}\right)}^{2}$$

where m,n indicate the height and width of the image.

In the process of unsupervised training, the size of similar sub-regions extracted is not fixed. In order to make the training stable and avoid the degradation of the size of the image, a penalty term for the area $L_{area}$ is added to the loss function in case that the area would be too small. Here, the $L_{area}$ is defined as:

(9) $$L_{area}={\left(1-d_{w}d_{h}\right)}^{2}$$

where $d_{w}$ and $d_{h}$ are the normalized scale parameters. The $L_{area}$ can force the network to search similar sub-regions in PMMWI and VI in a larger scope. To improve the stability and accelerate the training process, we preprocess the inputs based on Gaussian blur to make the contour of human smoother.

### 3.4. Detection and Synthesis Strategy

#### 3.4.1. Comprehensive Analyzer

**Figure 6:**
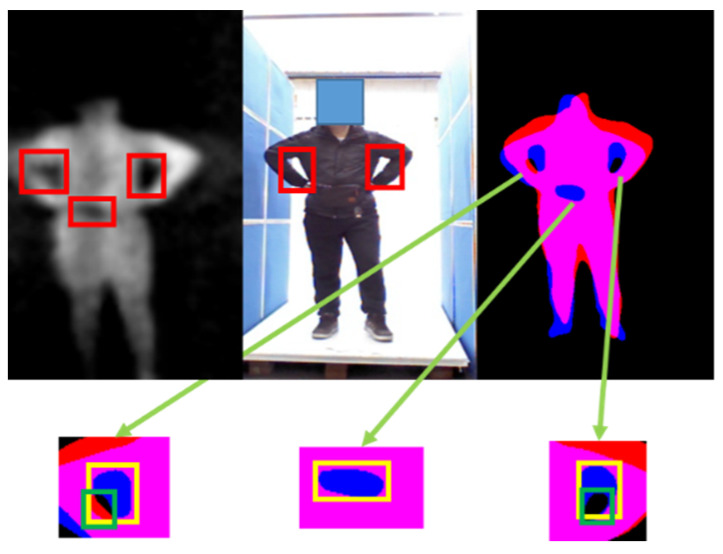
Illustration of our false alarm elimination strategy. The false alarms caused by the arm in the PMMWI have similar textural features to the threats, and it is difficult to be excluded only by the information from PMMWI. Using the registered VI to check the IOU of the corresponding area will help eliminate such type of false alarms. The yellow box in the second row is the suspicious area in PMMWI, and the green box represents the area extracted from the VI.

The detection and synthesis strategy is proposed for identifying the location and classification of threats. If a person carries a metallic threat during the security inspection, there is a cavity in PMMWI whereas the VI presents complete contour and the cavity could provide the location of the metallic threat. Meanwhile, non-metallic threats would be confirmed by the analysis of the contour of human body in PMMWI. A multi-fame synthetic strategy is applied then to integrate the information in each frame to obtain a comprehensive result, after which the region of metallic threats is extracted to be further identified. Here are the details:

1. Extract the connected components of segmented PMMWI and VI, then divide the image into three parts: the external background, the human body, and the suspicious area.

2. Concealed threats detection:

Metallic threats: compute the minimum area bounding box of the suspicious areas obtained from step 1, and then calculate the Intersection over Union (IOU) among these bounding boxes. If the IOU is greater than a threshold T, this area is considered as a false alarm, and it will be excluded. The remaining bounding box is deemed to be the metallic threats area. Details are shown in Figure 6.

Non-metallic threats: Extract the contour of human body in the segmented PMMWI and determine whether there is an abnormal area within it through the anomaly detection network. Then, calculate the corresponding bounding box.

3. Multi-frame synthetic strategy:

Accumulate the bounding boxes obtained in the third step for every 5 frames, where the inside is 1 and the outside is 0; and introduce continuity constraint to exclude the bounding boxes appearing less than three consecutive frames. Then, reserve the area with values greater than 3 as well as satisfying the continuity constraint and define it as a key frame. Calculate the IOU of threats area to human body area. Output the key frame with the largest IOU. Classify the metallic threats and visualize them on VI.

#### 3.4.2. Metallic Threats Classification Network

In this paper, the classification network is trained to recognize metallic knives and metallic guns. We employ the ResNet18 [41] for this task. The extracted threats region will be adjusted to 64×64, and the output is a 3-dimensional vector $v\in {\mathbb{R}}^{3\times 1}$. The vector is used to determine the category (metallic knives, metallic guns and unknown).

#### 3.4.3. Anomaly Area Detection Network

**Figure 7:**
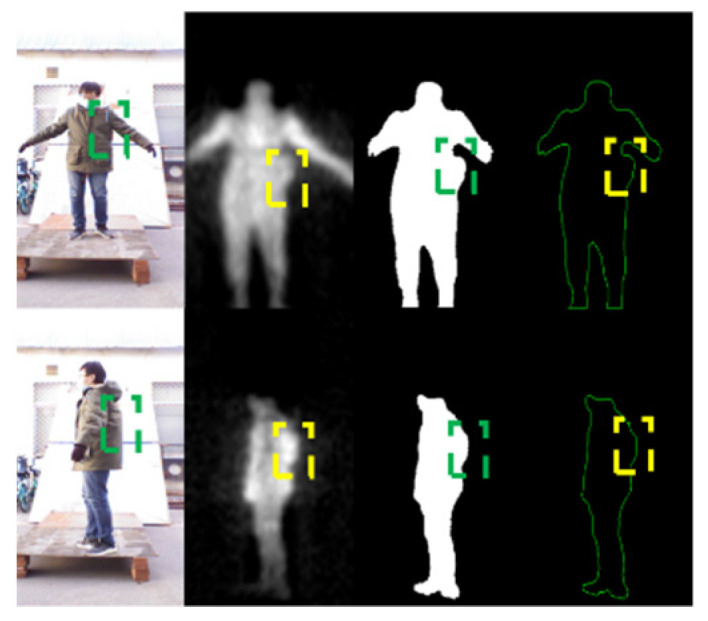
Illustration of PMMWI when a non-metallic threat is carried and the dashed boxes indicate the location of the threat.

For non-metallic threats, it is difficult to perform detection based on the grayscale in practical application. In Figure 7, volunteers carry non-metallic threats (gasoline) on the side waist and lower back. It is difficult to distinguish the threats from human body via grayscale. However, with a good penetrability, millimeter waves can present the non-metallic threats by highlighting the abnormal bulges on the images, according to which we can locate the non-metallic threats. In the fourth column of Figure 7, the contours of human body are fed into the network to learn the abnormal contour features caused by carrying threats and achieve the detection of non-metallic threats.

PointNet is originally designed to process 3D point cloud data and it is robust against incomplete shapes and target rotation. As an efficient feature extractor, it is adopted in this study. However, the input of original PointNet is the three-dimensional coordinates of the point cloud, while the contour information extracted in this study is two-dimensional. Therefore, to facilitate the detection of abnormal regions, the curvature features corresponding to the contour of the human body are concatenated to project to high dimensions. Therefore, the input of the network is $x\in {\mathbb{R}}^{b\times n\times 3}$, where b is the batch size during the training process and n refers to the numbers of points in the contour. The output of the network is $y\in {\mathbb{R}}^{b\times n\times 2}$, which represents the classification (normal or abnormal) of each point in the contour of human body, and we can find the area where the non-metallic is located according to the abnormal point.

## 4. Experimental Setup

### 4.1. Experiment Environment

**Figure 8:**
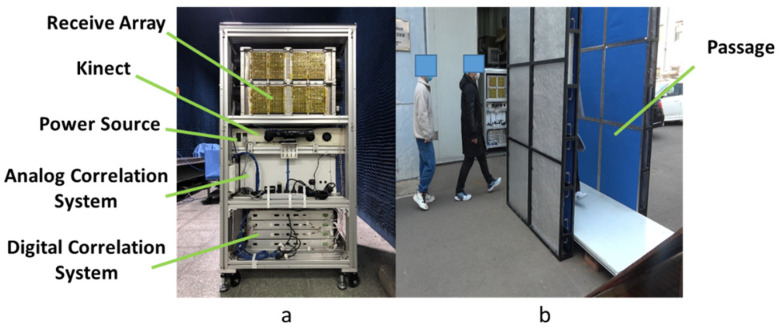
BHU-1024 including the main components (**a**) and experimental passage (**b**).

Our PMMWI Imager (BHU-1024) is shown in Figure 8a and related parameters are listed in Table 1. One analog PMMWI and two digital PMMWIs can be obtained from the imager per 40 milliseconds. VI is captured by Kinect V1. The size of PMMWI is 160 × 80 and 640 × 480 for VI. We install a passage in front of the imager as is shown in Figure 8b, and the imager conducts security inspection of the persons walking through the passage. The length of the passage is 1.6 m, the width is 1m, and the height is 2 m.

In this paper, we constructed our network with PyTorch and employed one PC with Intel i7-10700k at 3.8 GHz, 64 GB RAM and NVDIA GeForce RTX 3090 with 24 GB RAM.

### 4.2. The Collected Dataset

In this study, we took advantage of deep learning to improve the accuracy and robustness of detection. In order to achieve this goal, we collected and created a dataset to train the networks systematically.

**Figure 9:**
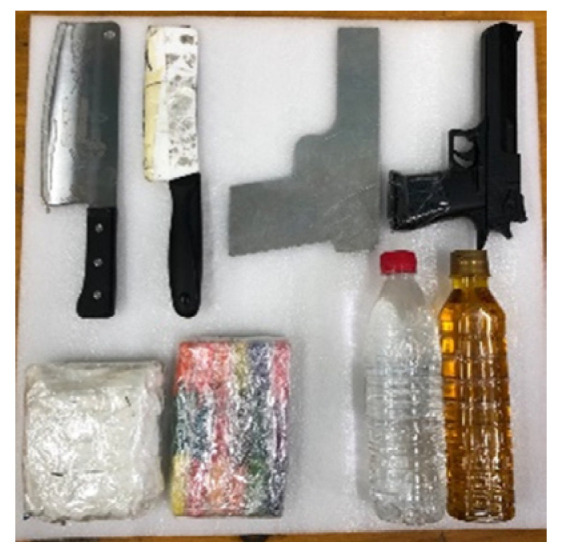
Simulated threats in our study. In the first row, from left to right, they are a metallic knife, a ceramic knife, a metallic gun, and a plastic gun. The second row is washing powder, plasticine, water, and oil.

**Figure 10:**
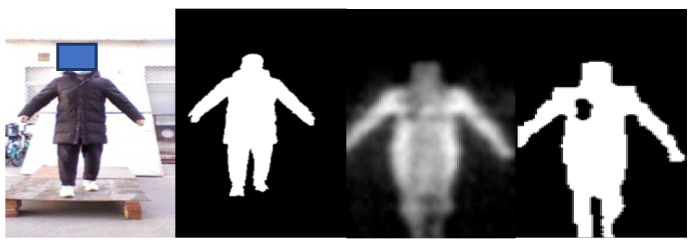
Example of segmentation dataset, including the paired VI and PMMWI, and the corresponding segmentation label.

**Figure 11:**
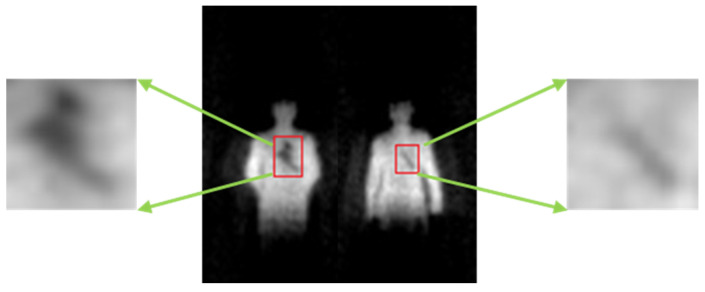
Example of classification dataset. We extracted the threats sub-region from PMMWI as training sample; the left side is the extraction region of the metallic gun, and the right side is the metallic knife.

**Figure 12:**
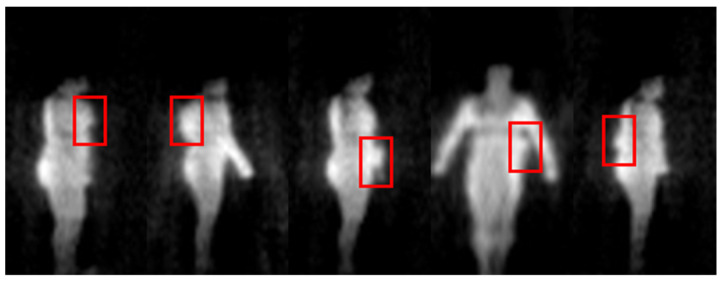
Example of anomaly detection dataset. The area contained in the bounding box is the abnormal area caused by the threats.

The PMMWI dataset was collected outdoors, and we collected images of volunteers walking through a straight passage in front of the device, and this was to simulate the real application scenarios. The threats were divided into 4 categories and 8 subcategories: metallic knives/ceramic knives, metallic guns/plastic guns, water/oil (simulating alcohol/gasoline), plasticine/washing powder (simulating plastic bomb/powder bomb), and are shown in Figure 9. 10 volunteers participated in the collection and these volunteers had different clothing types, figures and genders. The threats were fixed in 5 parts of the body: front chest, back, abdomen, lower back, and side waist. The VI and PMMWI were collected in pairs, the resolution of VI is 640×480 and the PMMWI is 160×80. After processing the data, we created three datasets: (1) the dataset for PMMWI semantic segmentation contains 11,021 images. (2) The dataset for metallic threats classification consists of 4627 images. (3) The dataset for anomaly detection of human body contour includes 5102 images. We present some examples of the dataset in Figure 10, Figure 11 and Figure 12.

### 4.3. Experimental Results and Discussion

#### 4.3.1. Image Fusion

To verify the performance of the proposed fusion network, three typical fusion methods are discussed: VIF-Net [42], DDcGAN [34]. VIF-Net adopts a CNN architecture for fusion; it mainly contains feature extraction and feature reconstruction modules. The network is trained by the proposed loss function for retaining features of source domain. While DDcGAN constructs a dual-discriminator conditional GAN for image fusion. It takes images of different resolutions as input and extract discriminative feature for reconstructing fused image. All these fusion methods are implemented based on public available codes, and the corresponding parameters are consistent with the original papers.

**Figure 13:**
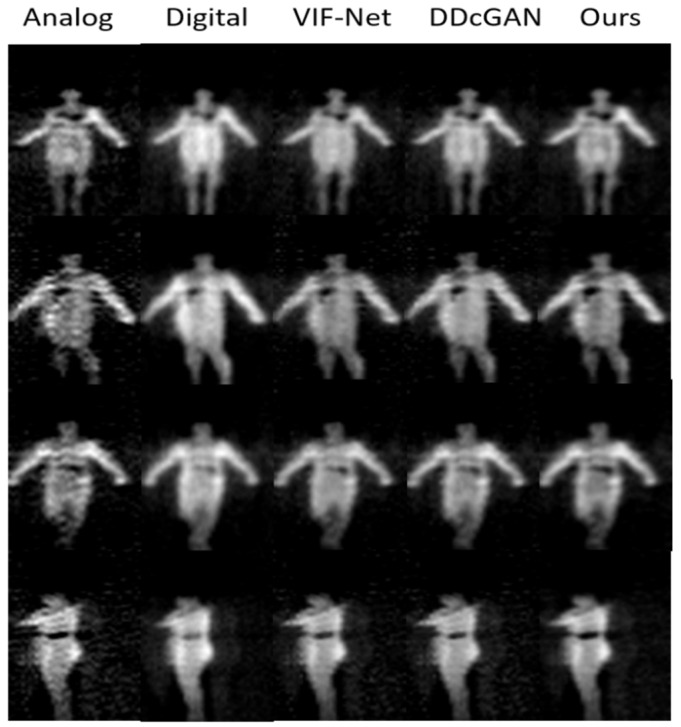
Experiment results of image fusion including four instances (each row). The first and second columns are the raw analog and digital PMMWIs, and the last three columns are the fusion images using three different methods.

The fused image is evaluated from qualitative and quantitative perspectives, which is the input to threats detection and also provides feedbacks for the operator. We applied Entropy (EN) [43], Peak signal-to-noise ratio (PSNR) [44] and Structural similarity index measure (SSIM) [45] to quantify the performance of different methods. The experiment results are shown in Figure 13. In addition, the quantitative comparison is listed in Table 2.

From Figure 13, we can observe that the threats in analog PMMWIs are more obvious for extracting features; however, due to the high sensitivity, more background noise is also introduced into the image. On the other hand, the human body in the digital PMMWI has smoother edge and more even texture. Compare the last three columns, the contrast of the fused image (the threats and the human body) generated by our method is higher than that produced by the other two methods. The results indicate that the network has effectively extracted the features which help locate the threats from the analog PMMWI. Meanwhile, it is obvious that the fused image has similar texture with the digital PMMWI. Moreover, compared with VIF-Net and DDcGAN, our method has reduced the noise of the fused image and highlights the threats. According to Table 2, our network achieves the highest PSNR and SSIM which indicate that the features from source images are preserved the best, especially the edges, texture, and contrast. At the same time, SSIM shows that the fused image maintains a high degree of similarity with the source image.

#### 4.3.2. Image Segmentation

In this section, we will discuss the improvement of the proposed separate batch normalization layers for learning process from multi-source data. There are three situations: (1) Network not shared: Two separate TernausNet are used to train on PMMWI and VI respectively, while the architectures of the two networks are same. (2) Network shared: Only one TernausNet is adopted, and all the parameters shared between PMMWI and VI. (3) Separate Batch Normalization: The proposed separation batch normalization layer is introduced in the encoder of TernausNet, it also employs one TernausNet with the layers in the network except the separation batch normalization layer are shared. We adopt *IOU* as the metric of network performance, it is formulated as:

(10) $$IOU=\frac{area\left(ROI_{seg\;}\cap^{\;}\;ROI_{label}\right)}{area\left(ROI_{seg\;}\cup^{\;}\;ROI_{label}\right)}$$

where $ROI_{seg\;}$ is the region of human body in the segmented image and $ROI_{label}$ is the related label. The network has better performance when the *IOU* is close to 1.

**Figure 14:**
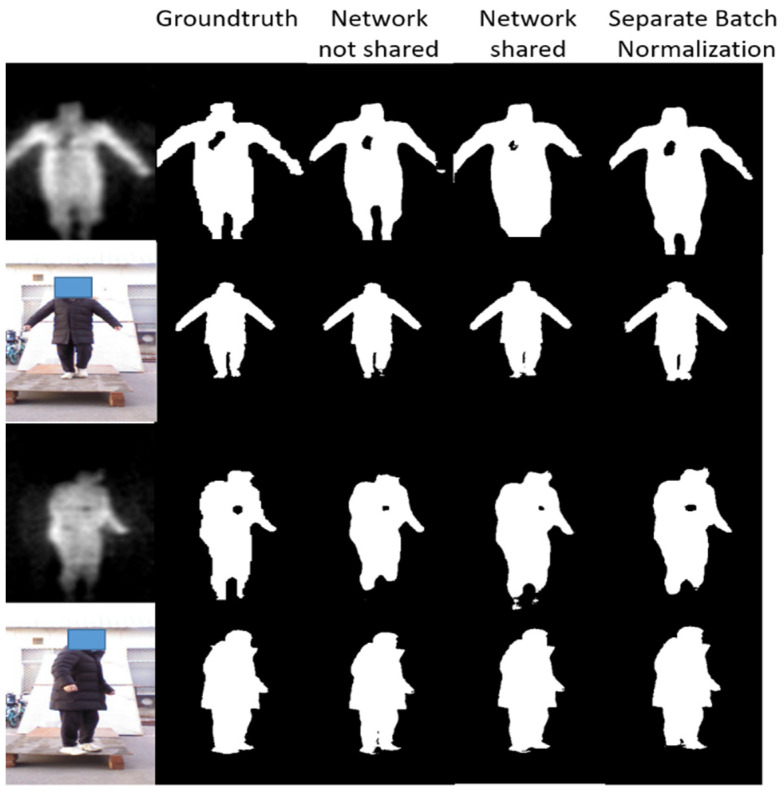
Experiment results of image segmentation. The first column consists of the raw VI and fused PMMWI, the second column is the corresponding groundtruth. The last three columns are the segment images under different networks.

The result of segmented images is shown in Figure 14 and Table 3 presents the performance of the proposed semantic segmentation network constructed with three strategies on the test set.

From Figure 14, the segmentation effect of network with separate batch normalization layers has been greatly improved compared with the network that fully shares the parameters. The contours of human body, the threats area and even the gaps in the legs can be completely and finely segmented. It can be concluded from Table 3 that the proposed separate batch normalization layer can improve the performance of network significantly compared with the fully shared network. Our segmentation effect is nearly the same as of two networks and the size of network is not changed.

#### 4.3.3. Image Registration

**Figure 15:**
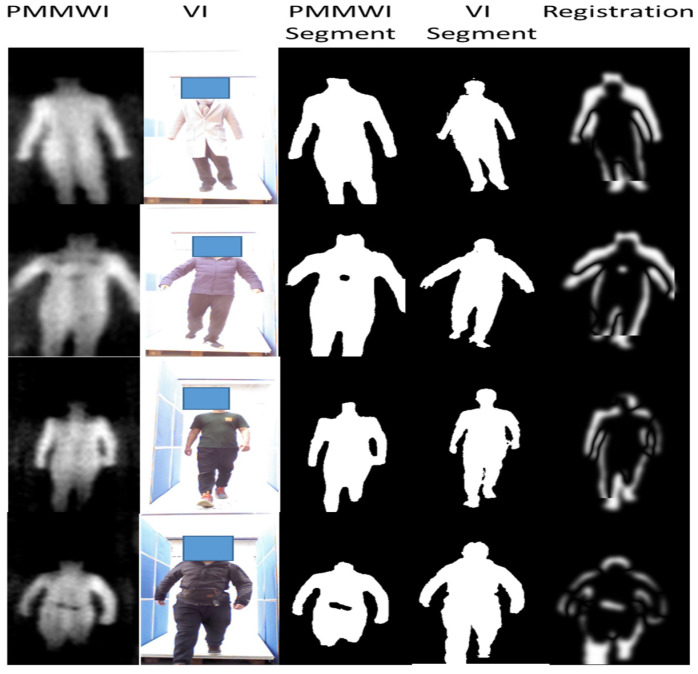
Experiment results of our registration network. The first and second columns are the raw PMMWIs and VIs, the third and fourth columns are the corresponding segmented images, and the last column presents the registration results.

The input of the registration network is the binary image after the segmentation network. The labeled dataset is utilized for training and verification of the registration network, while the output of the segmentation network is directly used when testing online. The experiment results are shown in the Figure 15.

In Figure 15, the correspondence of ratio and FOV between the original PMMWI and VI (the first and second columns) is the same as the corresponding segmented images (the third and fourth columns). From the third and fourth columns of Figure 15, the scales and FOVs of human contours in PMMWI and VI differ as the distance between the human and the device through the security inspection changes. Meanwhile, the ratio of the scale in PMMWI to that in VI is variable and the same for FOV. To evaluate the accuracy of registration, we subtract the registered PMMWI and VI pixel-wise, and subtracted pixel values can present the degree of similarity between two images. If the subtracted pixel value is close to 0, the two images share a high level of similarity. From the fifth column of the Figure 15, we can see that human body areas in PMMWI and VI are adjusted to a similar ratio. Even when the contour of the human body in PMMWI is incomplete (the fourth line of the Figure 15), the network can still find the best matching area from the complete contour in the corresponding VI and perform registration. We compared our method with the calibration-based method which measures the relationship of FOV between PMMWI and VI at several fixed points, and interpolates the conversion coefficient during test [32]. According to (10), the mean IOU of our method is 0.8673, and 0.7346 for calibration-based method; therefore, our method overcomes the shortage of the low accuracy of interpolation and achieves better registration effect. We can also observe that through registration, the concealed threats shown in PMMWI have been mapped to VI.

#### 4.3.4. Mental Threats Detection

**Figure 16:**
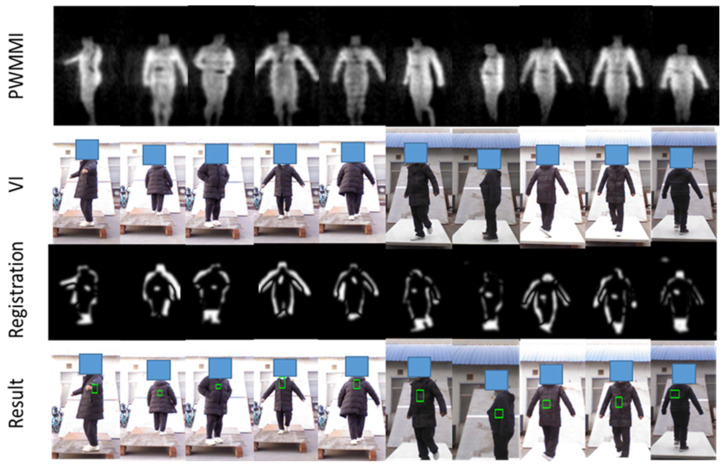
Location results of metallic threats based on multi-frame synthesis. The detected metallic threats are marked with a green box on VI.

To verify the detection algorithm for metallic threats, multiple volunteers carry metallic threats (metallic guns and metallic knives) in different positions and postures. We also present the result which is derived from the multi-frame synthesis strategy in the Section 3. The experimental results are illustrated in Figure 16.

**Figure 17:**
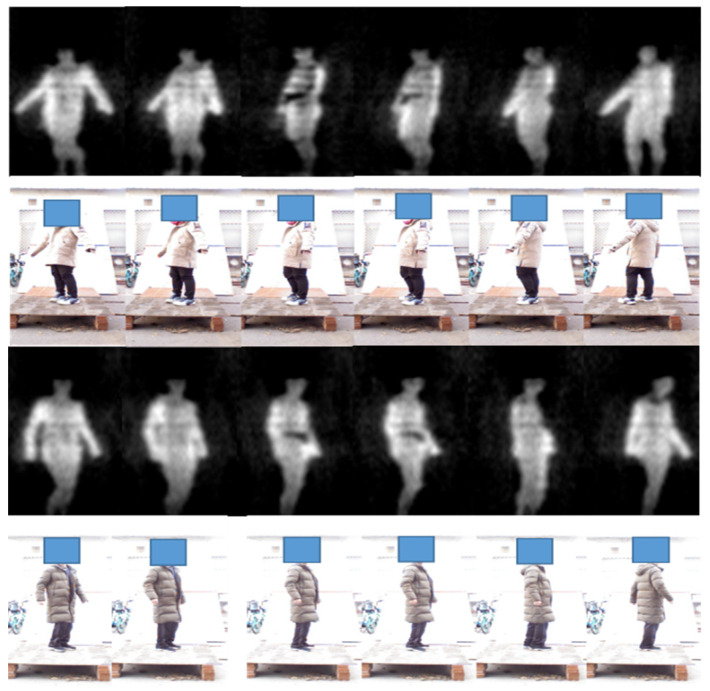
Examples of metallic threat detection under specific imaging angle.

From Figure 16, we can see that the threats under the clothes have been clearly displayed in the PMMWI. Comparing the second with the fifth column and the third with the fourth column, the threats can be accurately located on the waist and chest, which confirms that our registration network has achieved a high level of accuracy. It should be noticed that volunteers walk into two directions (forward or backward). Since, in the real application, we deploy two PMMWI imagers to collect PMMWIs in front of and behind human body simultaneously, the security inspection can be completed all-round and quickly. The results show that our method can effectively detect threats from two directions. Moreover, there is a significant advantage in our method: the inspection taking place during a walk which can improve the efficiency of security inspections greatly. Moreover, it is easier to capture the threats with the changing of imaging angle of threats. As illustrated in Figure 17, the threats carried by the two volunteers are held on the side waist. Under this circumstance, the threats cannot be shown in the PMMWI by the imager located at front or back; however, when the person is turning around, the threats can be captured with the proper imaging angle, then our method can perform the localization. This situation corresponds to the first and sixth columns.

After locating the threats, the types of threats are further classified. We evaluate the classification network on the test dataset and the classification confusion matrix is provided in Table 4.

From the confusion matrix, we can also calculate the rate of the accuracy of the classification and it is 92.52%. It is worth noting that if the contour of threats is incomplete or the localization is inaccurate, some metallic guns will be probably misclassified as metallic knives.

#### 4.3.5. Non-Mental Threat Detection

**Figure 18:**
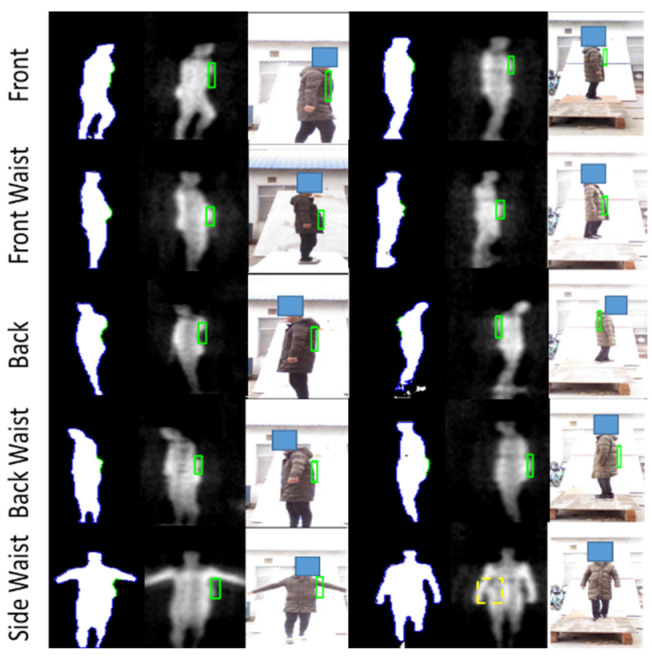
Visualized non-metallic threat detection of five different carrying positions. The first and fourth columns are the segmentation results of the abnormal area of human contour, and the green part is the abnormal area. The second and fifth columns mark the abnormal area on the raw PMMWI, and the third and sixth columns are the final detection results shown with VI.

For non-metallic detection, the input of body contour is obtained from the segmented PMMWI. During the test, the volunteers were also required to carry non-metallic threats and hold them in five locations and walk in the same way as in metallic detection. The test result is shown in Figure 18.

**Figure 19:**
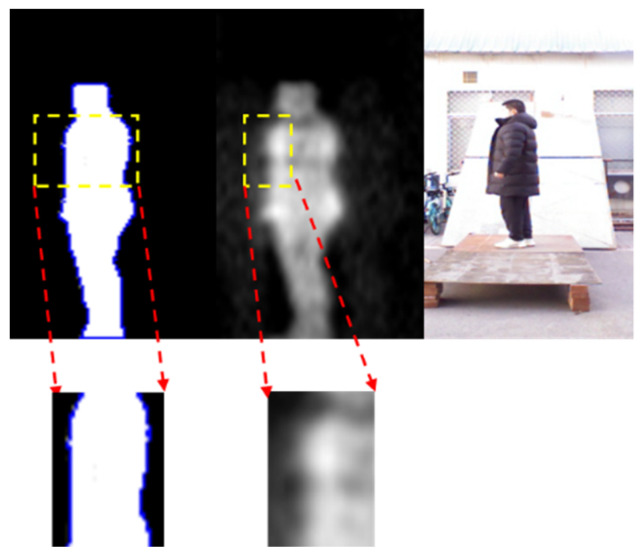
Example of a failure case, where the small size threats cannot provide sufficient contour information, and may disable the performance of anomaly area detection network.

It can be seen from first and second columns of Figure 18 that when the person carries threats, the body can be seen on PMMWI with obvious abnormal area due to a good penetrability of millimeter waves to clothing, and thus our method can effectively localize these areas. It is worthwhile to note that in the fifth row when the threats are placed on the side waist, raising arm will be more conducive to extracting the contour of this area, and in the fourth to sixth columns, the contour of the threats in the segmented human body cannot be extracted due to the interference of arm, which bring about incomplete contour of threats in PMMWI. However, compared with the detection of metallic threats, the detection of non-metallic threats only refers PMMWI to extract the abnormal areas from human body, and due to a low constraint of resolution of PMMWI, threats in small size may not be effectively extracted. Figure 19 demonstrates details for this situation, and it shows that small threats cannot be detected from the human body.

#### 4.3.6. Online Verification

Through offline test of each sub-module, the effectiveness of the algorithm is verified. We will integrate these sub-modules to verify the effectiveness of the algorithm under real application. In online test, 5 volunteers circulated through the passage and each person was required to take the test 50 times. We randomly selected two volunteers to carry designated types of threats and the threats were placed in the same location as in Figure 9. We use Recall and Precision as the evaluation criterion. Recall is the ratio of the number of found samples to the actual number of samples and it can measure all samples of a particular category. Precision is a metric that only identifies a specific category, reflecting the false alarm rate of the method. These criteria are given by:

(11) $$Precision=\frac{TP}{TP+FP}$$

(12) $$Recall=\frac{TP}{TP+FN}$$

The summary of consuming time for each module, the statistical detection results, and confusion matrix for metallic threat classification are given in Table 5, Table 6 and Table 7, respectively.

From Table 5, it can be observed that our method has a fast calculation speed and occupies less space. From the detection results in Table 6, we can see Recall and Precision of non-metallics threats are overall lower than that of metallic detection. This is because the process of metallic detection utilizes the information of both VI and PWMMI, while the non-metallic detection only refers to the information in PWMMI; moreover, the contour information is easier to be affected by noise due to a low image resolution, which makes its robustness decreased. The detection effect of metallic threats is weaker than that of non-metallic threats when the threats is located on the side waist, as the gray information in PMMWI only appears when the person turns around during the detection. The gray information is also easily disturbed by noise. Further study can improve the imaging algorithm in the future. According to Table 7, we can obtain the accuracy of the classification of metallic threats is 91.61% which is lower than that of test dataset.

To further validate the performance of the proposed method, we also compared our method with approaches given in other literature, including YOLOv3 [29] and HBPSNs-4 [32]. The YOLOv3 a typical one-stage detector, it treats the object detection as a regression problem, and directly obtains the bounding box, the confidence and the probabilities of being a certain category by taking whole image as the input. Its significant advantage is the trade-off between accuracy and detection speed, and has been applied in many real-time scenes. HBPSNs-4 also involves human body segmentation based on networks and complementary advantages of PMMW and VI, it trains two segmentation networks for PMMWI and VI, and the registration of PMMWI and VI is performed according to correspondence by off-line calibration. The relevant parameters of model are consistent with those in the original paper. Considering that these approaches only use the grayscale information of the PMMWI, we will focus on the detection of metallic threats. The experimental results are listed in Table 8.

A practical PMMWI imaging system needs to cope with the scene of large passenger flow, and the detection accuracy and false alarm rate are both important indicators. The YOLOv3 performs detection only relying on the PMMWI, which is a grayscale image. And the gray values of the concealed threats are similar to the values of background, so PMMWI as the only way of detection would produce false alarms. From Table 8, we can see that the complementary combination of PMMWI and VI can effectively reduce the false alarm rate of detection and improve the accuracy of the system. At the same time, our method uses the multi-source PMMWI fusion strategy to enhance the image quality and to further improve the efficiency of the detection.

## 5. Conclusions

Non-cooperative and fast security inspection system meets the requirement of the security inspection in large public place. In this paper, we designed and implement a real-time detection method which takes the advantages of multi-source PMMWIs and VIs to achieve a better detection for both metallic and non-metallic threats. We designed an image fusion network to obtain high-quality PMMWIs by comprehensively using digital and analog PMMWIs. A separate batch normalization layer is proposed and introduced into the existing semantic segmentation. We also present an image registration network based on unsupervised learning and the strategy for extracting similar sub-regions. In our method, the PMMWI and VI are synthetically utilized for high-precision segmentation of human body and locations of metallic threats, and through the accurate registration between PMMWI and VI, false alarm is further removed. In terms of non-metallic threats, which is tricky for PMMWI inspection system, we explored the contour feature of human body with deep neural networks and achieved the detection of large non-metallic threats. To fully utilize the sequential information between frames, a synthesizer based on each detection results was employed. The experiments demonstrate that our method outperforms the previous methods in terms of precision and recall, revealing that the fused high-quality PMMWI is essential to detect concealed threats. Meanwhile, the high-precision segmentation and adaptive registration helps accurate location and extraction of threat areas, and the multi-frame synthetic strategy further reduces the false alarms which is critical for practical application. We also show that our method achieves efficient detection for both metallic and non-metallic threats, providing a new solution for detecting hard samples (non-metallic threats) in PMMWIs.

The proposed method focused on real-time concealed threat detection from the human body in PMMW images during large passenger flows. Our method achieves better trade-off in terms of accuracy, speed, and computation resource; it should be beneficial for promoting the application of the PMMW security system. Due to the equipment limitations, the effect of non-metallic detection is imperfect when the detected threat is in small size. In the future, we will continue to explore the characteristics of non-metallic threats in the PMMWI, and it is necessary to use new types of information in the research to improve the detection capacity of non-metallic threats.

## Figures and Tables

**Table 1:** Specification of BHU-1024.

Height	1.6 m
Width	0.8 m
Frequency	32–36 GHz
Number of channels	1024
Imaging Resolution	4 cm@2 m
Temperature Resolution	1–2 K
Imaging Frame Rate	25 FPS
Observation Distance	1–4 m
Number of Pixels *	80 × 160
Observation Range *	1m × 2m

* horizontal direction × vertical direction.

**Table 2:** Quantitative results of different fusion network.

Network	EN	PSNR	SSIM
VIF-Net	5.595	20.633	0.868
DDcGAN	6.203	20.547	0.867
Ours	6.158	22.350	0.901

**Table 3:** Performance of Different Network Architectures.

Network	IOU	Parameters
Network not shared	0.9276	141.74 M
Network shared	0.9230	70.87 M
Separate Batch Normalization	0.9271	70.88 M

**Table 4:** Confusion Matrix of Threat Classification on dataset.

	Ground Truth	Metallic Gun	Metallic Knife	Other
Prediction				
Metallic Gun		219	5	7
Metallic Knife		29	114	4
Other		2	10	372

**Table 5:** Time Cost of each module in our method.

	Time	Network Size
Image Fusion	10.74 ms	15.24 M
Image Segmentation	13.77 ms	70.88 M
Image Registration	11.28 ms	40.24 M
Detection (non-metallic)	12.32 ms	22.26 M
Detection (metallic)	13.77 ms	43.74 M

**Table 6:** Online Detection Result.

Position	Metallic (Recall)	Non-Metallic (Recall)	Average (Recall)	Metallic Precision)	Non-Metallic (Precision)	Average (Precision)
Front Chest	95%	83.33%	89.17%	91.34%	90.91%	91.13%
Back	97%	84.33%	90.67%	93.26%	92.63%	92.95%
Abdomen	94%	80.66%	87.33%	94%	92.06%	93.03%
Lower Back	95%	84.33%	89.67%	93.14%	91.01%	92.07%
Side Waist	84%	85.33%	84.67%	94.38%	90.78%	92.58%

**Table 7:** Confusion Matrix of Threat Classification in Online Test.

	Ground Truth	Metallic Gun	Metallic Knife	Other
Prediction				
Metallic Gun		210	3	0
Metallic Knife		12	216	0
Other		10	14	0

**Table 8:** Performance Comparison among Different Methods.

Algorithm	Recall	Precision	FPS
YOLOv3	89.34%	89.03%	34
HBPSNs-4	87.37%	90.57%	18
Ours	90.30%	92.35%	24

## Data Availability

Not applicable.
